# Supplementary figures and images for: Predicting malaria outbreak in The Gambia using machine learning techniques
Source: PLoS One. 2024 May 16;19(5):e0299386. doi: 10.1371/journal.pone.0299386 (PMC11098333; doi:10.1371/journal.pone.0299386)

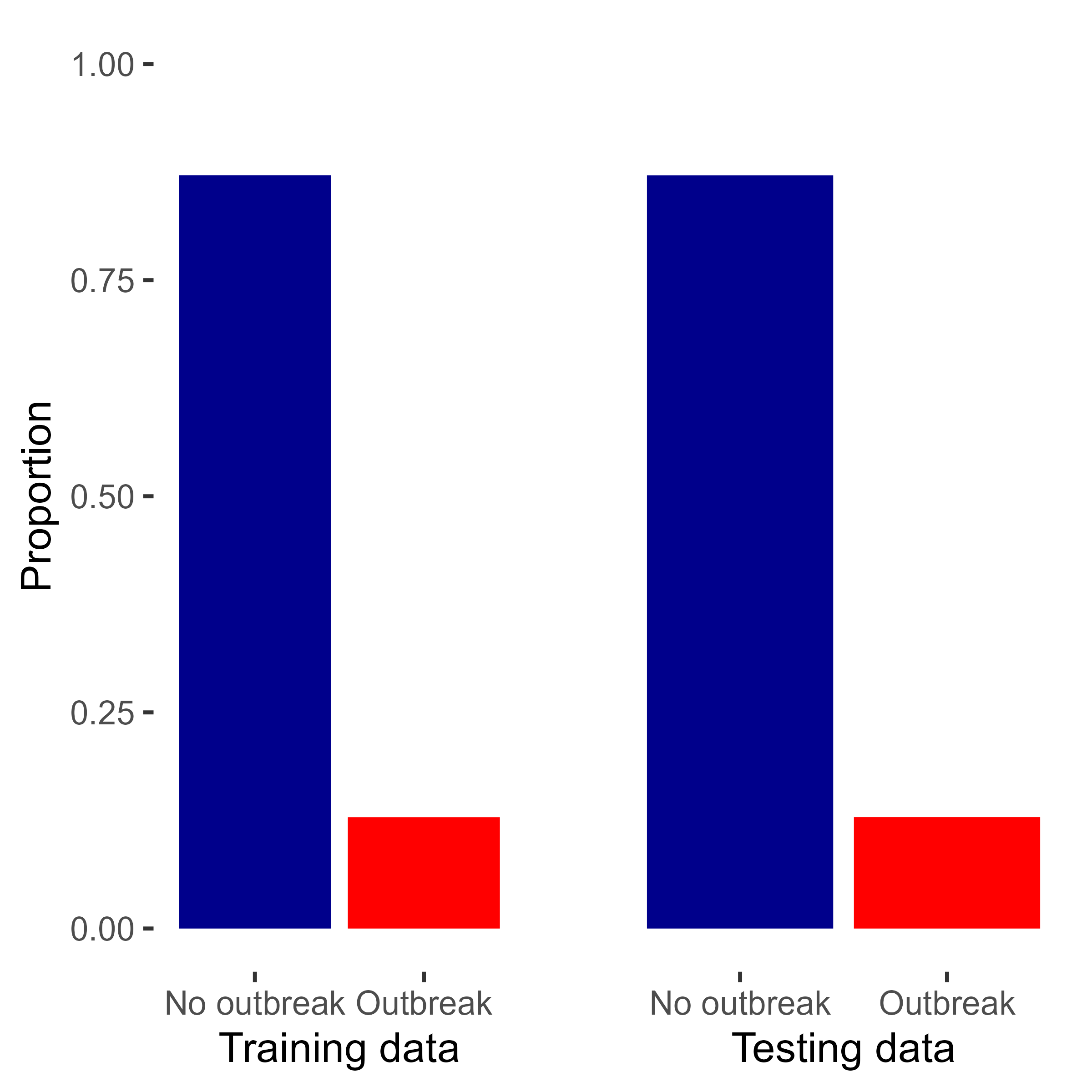

Supplement: S1 Fig — (TIFF) [file pone.0299386.s001.tiff]
